# Supplementary material for: Statin Therapy and the Risk of Viral Infection: A Retrospective Population-Based Cohort Study
Source: J Clin Med. 2022 Sep 24;11(19):5626. doi: 10.3390/jcm11195626 (PMC9571401; doi:10.3390/jcm11195626)
Supplement: Supplementary file 1 [file jcm-11-05626-s001.zip › jcm-1914511-supplementary.pdf]

Supplemental Table S1. ICD-9-CM codes for diseases

ICD-9-CM codes	Diagnosis
272	Hyperlipidemia
401-405	Hypertension Diabetes
250	Rheumatoid disease
714	Alcohol-related disease
291, 303, 305, 571.0, 571.1, 571.2, 571.3,	
790.3, V11.3	Asthma
493	Transplantation
V420, V421, V426, V427, V4281,	
V4282, V4283, V4284, V4289, 99681,	
99682, 99683, 99684, 99685, 99686	Chronic liver disease
571	CKD or ESRD
585, 586, 588.8-588.9	
	COPD
491, 492, 496	HIV
042-044, 7958, V08	Cancer
140-208	CHF
428	Stroke
430-438	
071-079, 045-049, 055-056, 060-066, 460	Viral infection
